# Opioid Treatment Deserts: Concept development and application in a US Midwestern urban county

**DOI:** 10.1371/journal.pone.0250324

**Published:** 2021-05-12

**Authors:** Ayaz Hyder, Jinhyung Lee, Ashley Dundon, Lauren T. Southerland, David All, Gretchen Hammond, Harvey J. Miller

**Affiliations:** 1 Division of Environmental Health, College of Public Health, The Ohio State University, Columbus, OH, United States of America; 2 Translational Data Analytics Institute, The Ohio State University, Columbus, OH, United States of America; 3 Department of Geography and Environment, Faculty of Social Science, Western University, Social Science Centre, London, ON, Canada; 4 Department of Emergency Medicine, College of Medicine, The Ohio State University, Columbus, OH, United States of America; 5 Founder and CEO, Mount Ethos, Seattle, WA, United States of America; 6 College of Social Work, The Ohio State University, Columbus, OH, United States of America; 7 Center for Urban Regional Analysis, The Ohio State University, Columbus, OH, United States of America; 8 Department of Geography, The Ohio State University, Columbus, OH, United States of America; Columbia University, UNITED STATES

## Abstract

**Objectives:**

An Opioid Treatment Desert is an area with limited accessibility to medication-assisted treatment and recovery facilities for Opioid Use Disorder. We explored the concept of Opioid Treatment Deserts including racial differences in potential spatial accessibility and applied it to one Midwestern urban county using high resolution spatiotemporal data.

**Methods:**

We obtained individual-level data from one Emergency Medical Services (EMS) agency (Columbus Fire Department) in Franklin County, Ohio. Opioid overdose events were based on EMS runs where naloxone was administered from 1/1/2013 to 12/31/2017. Potential spatial accessibility was measured as the time (in minutes) it would take an individual, who may decide to seek treatment after an opioid overdose, to travel from where they had the overdose event, which was a proxy measure of their residential location, to the nearest opioid use disorder (OUD) treatment provider that provided medically-assisted treatment (MAT). We estimated accessibility measures overall, by race and by four types of treatment providers (any type of MAT for OUD, Buprenorphine, Methadone, or Naltrexone). Areas were classified as an Opioid Treatment Desert if the estimate travel time to treatment provider (any type of MAT for OUD) was greater than a given threshold. We performed sensitivity analysis using a range of threshold values based on multiple modes of transportation (car and public transit) and using only EMS runs to home/residential location types.

**Results:**

A total of 6,929 geocoded opioid overdose events based on data from EMS agencies were used in the final analysis. Most events occurred among 26–35 years old (34%), identified as White adults (56%) and male (62%). Median travel times and interquartile range (IQR) to closest treatment provider by car and public transit was 2 minutes (IQR: 3 minutes) and 17 minutes (IQR: 17 minutes), respectively. Several neighborhoods in the study area had limited accessibility to OUD treatment facilities and were classified as Opioid Treatment Deserts. Travel time by public transit for most treatment provider types and by car for Methadone-based treatment was significantly different between individuals who were identified as Black adults and White adults based on their race.

**Conclusions:**

Disparities in access to opioid treatment exist at the sub-county level in specific neighborhoods and across racial groups in Columbus, Ohio and can be quantified and visualized using local public safety data (e.g., EMS runs). Identification of Opioid Treatment Deserts can aid multiple stakeholders better plan and allocate resources for more equitable access to MAT for OUD and, therefore, reduce the burden of the opioid epidemic while making better use of real-time public safety data to address a public health epidemic that has turned into a public safety crisis.

## 1. Introduction

In 2017, Ohio had the 2^nd^ highest drug overdose mortality rate in the nation (46.3 deaths per 100,000 population), with the death rate rising from 2013 to 2017 [1, 2]. While efforts are underway at the local, state, and national level to reduce opioid prescriptions and increase availability of the antidote naloxone (brand name Narcan), less attention has been given to addressing barriers faced by individuals seeking treatment after suffering an opioid overdose event (OUD) [3, 4]. In Ohio, methadone can only be dispensed by a certified Opioid Treatment Program (OTP), prescribing buprenorphine can only be done by a licensed medical provider with a U.S. Department of Justice Drug Enforcement Administration registration and a buprenorphine waiver and naltrexone can only be prescribed by any licensed medical provider [5]. Since 2011 changes in Medicaid have contributed to increasing the number of substance abuse disorder (SUD) treatment providers in Ohio but medical facilities and other specialized settings including OTPs, residential substance abuse facilities, or outpatient behavioral health organizations have been slow to open in certain parts of Ohio, including Franklin County, Ohio [5].

Those who desire treatment after an opioid overdose event often encounter additional significant barriers to accessing services. Addressing barriers related to accessing treatment for persons with SUD—specifically OUD—is the focus of this paper. Even more specifically we focus on accessing treatment and recovery as depicted by the Cascade of Care model of OUD [6]. The encounter with a healthcare provider just after an opioid overdose event presents an opportunity to intervene as a part of the treatment cascade, which is one component of the Cascade of Care model for OUD, by engaging with the patient and presenting options for accessing services [7]. Accessing healthcare and behavioral health services must be considered comprehensively, including the structural features of the healthcare and behavioral health system and the urban environment, individual features that can enable or be barriers to care, and process factors [8]. For example, a person may have potential access to a clinic within walking distance but if that person does not have the correct insurance to access that clinic, it is still inaccessible [9]. Access-related barriers to healthcare can be subdivided into five categories: availability, affordability, accommodation, accessibility, and acceptability [9, 10]. The availability (i.e., number of clinics) and accessibility (i.e. ability to get to clinic) of evidence based treatment for OUD (i.e. medication assisted treatment or MAT) are major barriers to addressing the rising burden of the opioid epidemic [11, 12]. Not only have the barriers to accessing treatment been given little attention, they have also been largely focused on the individual’s ability to overcome the barriers, rather than examining larger structural situations that help perpetuate the barriers in the first place. For example, the decisions for where to locate treatment services, what populations to serve, and what services to offer, can create multiple barriers for individuals that they then must work to overcome in order to gain access. Patient-centeredness is a feature of healthcare delivery organizations that captures this exact issue [8] and healthcare organizations that create a culture of patient-centeredness are positioned to prioritize needs of patients across multiple dimensions of access to healthcare services [13].

Furthermore, access in the health geography literature is delineated into spatial (e.g., mode of transportation, distance) and non-spatial accessibility (e.g., insurance, type of services offered) and realized and potential stages of access [14]. The focus of this study is on identifying areas with low spatial accessibility at the point where the patient is introduced to the healthcare system, which is how we label this idea throughout the study. Our conceptualization of potential spatial accessibility considers the time travelled to a treatment provider by anyone who suffers an opioid overdose event and may need access to OUD treatment regardless of their grouping by social, economic, demographic or clinical factors [15]. Previous studies have identified several barriers to accessing OUD treatment, such as convenience of travel time [16], availability and capacity of treatment providers [17, 18] and mode of transportation [19]. Building on these previous findings and leveraging the spatial turn in health research [20], we present a method for reconsidering the barriers that exist and addressing them through geographic considerations.

In addition to geographic considerations, there are structural barriers to consider as well. Structural barriers may perpetuate and amplify racial disparities in opioid overdose events [21] and inequities in access to OUD treatment providers. Some of these barriers include structural racism [22], biases in data collection processes for race/ethnicity data in electronic health records [23] and mistrust of the healthcare system by people of color [24–26]. The long-term and historical nature of some of these barriers manifests within geographic analysis of OUD because of racial residential segregation and “not in my back yard” mentality [27], which may affect location of SUD treatment providers [28]. Therefore, it is imperative to study racial differences in relation to potential spatial accessibility because identification of such differences may provide the evidence needed to take action towards eliminate racial disparities in opioid overdose events and eliminate racial inequalities in access to OUD treatment providers.

Based on these two considerations (spatial and structural barriers), our first hypothesis is that geographic areas exist with low potential spatial accessibility to OUD treatment and recovery services. We call this concept and areas that meet this hypothesized criterion, an *Opioid Treatment Desert*. Our second hypothesis is that potential spatial accessibility to OUD treatment and recovery services varies by race [29]. While the first hypothesis is not a new hypothesis, prior studies have quantified these disparities at the state or county wide levels only which does not necessarily assist local health departments and agencies on the front line of the opioid epidemic [30]. Prior studies have also studied the association between individual- and neighborhood-level covariates and spatial patterns of opioid overdose events at the sub-county level but have not addressed potential spatial accessibility to OUD treatment providers [31, 32].

Access within a county can vary considerably for the individuals living in that area. Identification of local barriers may assist in planning the response and optimal allocation of limited local resources to reduce the burden of the opioid epidemic. Also, identifying areas with spatial access to treatment providers using Geographic Information Systems (GIS) and a treatment provider database [33] is not enough because such an approach only identifies the travel-time based “market area” for treatment providers but not the overlap in demand for treatment. By “demand” here we refer only to those individuals who may be seeking access to treatment because treatment is not always clinically indicated for everyone seeking access to treatment. Therefore, using EMS data, which is not a perfect indicator but a good surrogate for demand, sets our study apart from others that have mainly focused on the supply side from the perspective of healthcare organizations. In other words, our use of EMS data and conceptualization of an Opioid Treatment Desert considers both the “market area” or supply for treatment from the healthcare service provider/organization’s perspective and the demand for treatment from the individual perspective. This exploratory study into the existence of Opioid Treatment Deserts and racial differences in potential spatial accessibility for OUD treatment and recovery services uses data from Columbus, Ohio (in Franklin County, Ohio) to demonstrate the concept of Opioid Treatment Deserts and discusses the concept’s practical implications for treatment from multiple perspectives, including public health, emergency medicine and social work.

## 2. Material and methods

This study was approved by the Ohio State University Biomedical Sciences Institutional Review Board and deemed to pose only minimal risk to human subjects (Approval # 2017H0220).

### 2.1 Study area

The spatial extent of the study area was the administrative boundary of Franklin County, Ohio, which includes the City of Columbus and the Greater Columbus area, with a population of just over 1.3 million people in 2019 [34]. Since 2010, the population of Franklin County grew by 13% [34]. Franklin County residents are served by multiple Emergency Medical Services (EMS) agencies with geographically distinct service areas (see S1 Fig). While the Columbus Fire Department has the largest service area in the county, several EMS agencies serve townships, villages and outlying suburbs of Columbus, OH. The study area for this analysis was the EMS jurisdiction of the Columbus Fire Department, which covers approximately 66% of the population in Franklin County, OH. Using a single EMS agency’s data, which is also expansive in terms of its spatial coverage (S1 Fig) of Franklin County, allowed us to reduce biases associated with merging data and using different definitions for what is considered an opioid overdose event.

### 2.2 Data collection

#### 2.2.1 Opioid overdose data (EMS)

*We used data from electronic patient care records compiled by the Columbus Fire Department as a* proxy for opioid overdose events based on the following criteria, which included a specific time period, list of chief complaints and impression (see below), and administration of specific medication. We requested patient-level data on all runs that occurred between 1/1/2008 and 12/31/2017, inclusive, but only data from 2013 to 2017, inclusive, was used in the final analysis because treatment provider data were consistently available for that time period only. Exclusion criteria were age <18 years old and not meeting the criteria for an opioid overdose event (see below). These data included fatal and non-fatal opioid overdose events.

The criterion used by the Columbus Fire Department for identifying an opioid overdose event was as follows: a paramedic run was classified as an opioid overdose event if 1) naloxone was administered by EMS/law enforcement/fire fighter **and** 2) impression or chief complaint was one of the following: opioid related disorders, substance abuse, drug abuse, poisoning/drug ingestion, cocaine related disorders, altered mental state, cardiac arrest. Since cardiac arrest may not always indicate an opioid overdose event we excluded individuals who presented with cardiac arrest only even if naloxone was given to the individual. Practically, this meant that we included individuals who presented with cardiac events and one of the following opioid related disorders, substance abuse, or drug abuse and had been administered naloxone. We requested the following variables from the Columbus Fire Department: scene address, location type of scene (i.e., a categorical description of the type of place, such as residence, street, parking lot), age, race, impression, chief complaint, disposition, destination and naloxone administration.

#### 2.2.2 Treatment provider data

Data on names and location of OUD treatment and recovery service providers was compiled from multiple sources including online treatment locators [35, 36], historical records from the National Directory of Drug and Alcohols Abuse Treatment Programs (NDAATP) [37–41], current resource guides compiled by the Franklin County Alcohol, Drug and Mental Health (ADAMH) Board and local public health departments (e.g., Columbus Public Health, Franklin County Public Health). Other studies have used similar approaches to identify OUD treatment providers for studying access to treatment [42]. In Ohio, local ADAMH Boards certify and license alcohol, drug and mental health providers in each county. The initial list that we compiled from the sources listed above contained 112 providers in Franklin County, OH. We included all providers in Franklin County, OH because we assumed that patients may seek treatment at any provider in the county regardless of where they suffer an overdose in the county. We filtered this list to keep only treatment facilities that offered Medication-Assisted Treatment (MAT) for OUD and that were operational from 2013 to 2017, inclusive. We used information from online treatment locators to identify facilities offering MAT (Buprenorphine, Methadone and Naltrexone) for OUD.

In our study, a treatment provider was included in the final analysis if they met the following conditions for OUD treatment: 1) used Buprenorphine, Methadone or Naltrexone for treatment of OUD based on information provided in the SAMSHA online treatment provider database, *or* 2) used Methadone, Suboxone or Vivitrol for treatment of OUD based on information provided in the ADAMH database. After applying this condition, 60 treatment providers remained from the 112 previously identified from multiple lists. We used the annual directory of treatment programs for Ohio from the NDAATP to check that each OUD treatment facility was in continuous operation from 2013 to 2017. Providers had to be listed in the directory for each of the five years in order to be included in the analysis. The final number of treatment providers included in the analysis was 22 providers. Some of these providers focused on treatment for men only, women only, and adolescents but, for this exploratory study, we did not verify the accuracy of this information for each provider across all years of operation.

### 2.3 Outcome measures

#### 2.3.1 Spatial accessibility measure

We geocoded the scene address of each opioid overdose event [43]. Addresses were geocoded using Geographic Information Software (GIS) software ArcGIS [44]. Geocoded addresses with a match score, which measures accuracy of the scene address to an address in the geocoding database, of greater than 75 (out of 100) were included in the final analysis. Addresses matched to zip code centroids were identified, manually corrected and geocoded again to check if they were geocoded to a street address. Some addresses contained unit/apartment numbers and spelling mistakes that led to lower match scores. These addresses were manually corrected for errors, where possible, and re-geocoded to match a known street address. We used the geocoded addresses for calculating potential spatial accessibility (see below).

For each opioid overdose event, we calculated how long it would take to travel (in minutes) by car or bus (public transit) from the overdose event location to the closest OUD treatment provider. Travel time was our primary measure of spatial accessibility and distance travelled was a secondary measure of spatial accessibility. We preferred travel times rather than distance travelled because travel times are easier to interpret and its calculation takes into account road speed limits as well as spatial (e.g., routes, stops) and temporal constraints (e.g., schedules, frequency) imposed by public transit services. Speed limits and transit operation data were available from OpenStreetMaps [47] and General Transit Feed Specification (GTFS) [45], respectively. We converted travel times to distance travelled for the purpose of visually categorizing areas as having low/high spatial accessibility. We did not directly calculate distance travelled because such a calculation would not have into account road speed limits and transit operation constraints, which are likely to affect potential spatial accessibility in urban areas that were included in our study area. We did not use the commonly known the Two-step Floating Catchment Area (2SFCA) method [46] for calculating spatial accessibility because we knew the exact location of both the origin (i.e., patient who suffered from an overdose event) and destination (i.e., treatment provider) for calculating potential spatial accessibility.

Travel times were calculated using a combination of Python packages (ArcPy, OD Matrix and Closest Facility solvers, Add General Transit Feed Specification (GTFS) to a Network Dataset tool) for ArcGIS Desktop (version 10.2.2), OpenStreetMaps [47], General Transit Feed Specification data [45] including detailed transit route and schedule information from the local transit authority (Central Ohio Transit Authority), and sidewalk network data from the local metropolitan planning organization (Mid-Ohio Regional Planning Commission). For transit travel time calculations, we assumed an arrival time of 9:00 AM on Wednesday. This time of day and day of week was chosen because it represents the typical weekday public transit schedule and considers that dosing times for those on MAT and individual/group counseling sessions typically start early in the morning at most treatment providers in Franklin County.

We used the full latitude and longitude coordinates for calculating travel times. After performing this calculation, we binned the travel times for visualizing travel times on a map (see section 2.3.2). This was necessary in order to meet the requirements for data privacy as outlined in our data use agreements with Columbus Fire Department. We used the R package osmdata to obtain the base map and data from OpenStreetMap [47] and OpenStreetMap Foundation for all maps.

#### 2.3.2 Classification of Opioid Treatment Deserts

Opioid Treatment Deserts were identified using a two-step process. First, we estimated travel time to the closest treatment provider for each opioid overdose event (see section 2.3.1). Second, we binned each opioid overdose event into a two dimensional grid by truncating the geocoded latitude and longitude value to four significant digits. By truncating coordinates in this manner, there was an appearance of a loose grid when the modified coordinates are overlaid a base map of the study areas and a square symbol was used to map the binned value. We calculated the average estimated travel time for each bin and classified the bin as low or high spatial accessibility based on the mode of transportation-specific threshold value.

As a sensitivity analysis, we used two different thresholds for each mode of transportation to classify high or low spatial accessibility. We also calculated travel time to closest treatment provider for EMS runs where scene location type was classified as “Home/Residence” only and for EMS runs where the individual’s race was listed as Black or White in the electronic patient care record. Note that EMS personnel categorized individuals into standard race categories although the patient may self-identify as a different race. We restricted the analysis to individuals with race listed as Black or White only because the next most common race, which was Hispanic, was less than 1% of the total patient population included in the analysis.

For travel by car, areas were identified by high or low spatial accessibility if the distance travelled by car was less than or equal to 1 mile or greater than 1 mile. We set the threshold to 1 mile because previous research [48] has shown that the probability of entering and remaining in treatment for a complete cycle as assigned by one’s level of care for OUD decline as much as 50% when patients lived more than a mile away from the treatment facility. Since we calculated potential spatial accessibility in minutes, we converted minutes travelled into distance travelled in order to use the 1-mile threshold. We also performed this conversion because it allowed us to compare our results with findings from the peer-reviewed literature [48]. To perform the conversion, we assumed travel speeds by car in a city of between 25 and 35 miles per hour (mph). As a result, it would take on average 2 minutes to travel one mile by car (average of 25mph and 35 mph is 30mph or 2 minutes per one mile). We also used a threshold of 2 miles by car as a sensitivity analysis because it was twice as much as the results reported in the main analysis. For travel by public transit, the main results are presented using a 30-minute threshold, which is a common threshold used in the accessibility literature for public transit [49, 50]. Our main results assumed a 30-minute threshold and supplementary results assumed a 15-minute travel time threshold for travel time by public transit. In summary, areas were classified as Opioid Treatment Deserts in the Columbus Fire Department jurisdiction if the closest treatment provider was spatially accessible based on the given threshold (1 mile away or more by car and 30 minutes travel time or more by public transit). We did not account for the number of overdose events in the analysis under the assumption that access to treatment should be available to everyone irrespective of the burden of the epidemic in their immediate surrounding [15]. Another reason for not accounting for number of overdose events is that we defined an Opioid Treatment Desert based on potential spatial accessibility as measured by travel time from an opioid overdose event as opposed to realized spatial accessibility. Realized spatial accessibility would explicitly take into account treatment provider capacity and number of patients seeking OUD treatment services but we did not use this measure because we did not have information on treatment provider capacity.

### 2.4 Statistical analysis

Descriptive statistics were generated for gender, race, destination (hospital) and scene location type. We used a chi-square test (alpha = 0.05) to determine if there were differences in age, gender and location by race. We used heatmaps to show the spatial and temporal evolution of the opioid epidemic in Franklin County and spatial differences by race and gender. The heatmaps were created using two-dimensional kernel density estimation via the ggplot package in R. We set the number of bins to 25 and left all other values in the kernel density estimation function to default. We used kernel density estimation for the purpose of visualizing the spatiotemporal dynamics and the association of demographic factors for opioid overdose events while preserving patient privacy [51]. We constructed maps of the study area showing areas that are Opioid Treatment Deserts based on spatial accessibility (high or low). Even though we calculated travel time to providers by type of MAT offered (e.g., Methadone, Buprenorphine, Naltrexone) we did not use this metric to identify Opioid Treatment Deserts because treatment service data was not consistently available for each type of MAT. Instead, we reported statistics on travel times to treatment providers by type of MAT offered and by race. We used a two-sided t-test (alpha = 0.05) to determine whether the estimated travel time for each MAT treatment type differed by race. R code for creating maps and conducting the statistical analysis is provided in the S1 Data. We used R (ggmap package) to create all maps. All statistical analyses were conducted using the R software (The R Foundation) [52].

## 3. Results

We initially received data on 11,901 opioid overdose events that took place in Franklin County, OH from 2008 to 2017, inclusive, from multiple EMS agencies operating in the county. Of these 11,304 events (95%) were successfully geocoded and 10,341 events were in Columbus Fire Department service area. From 2013 to 2017, there were a total of 6,929 opioid overdose events (67% of geocoded events in Columbus Fire Department service area) that were included in the final analysis.

For the overall sample (N = 6,929), most events occurred among 26–35 years old (34%), individuals who were male (62%) and White adults (56%) (Table 1). Over half of the opioid overdose events were responded to by paramedics at a residential type location (64%) such as, home, apartment, or condo, but streets/highways were also common location types (17%) (Table 1). In terms of age, Black individuals tended to be slightly younger than White individuals who experienced an opioid overdose event. Also, White adults (68%) tended to be found in Home settings more often Black adults 60%). Black adults (24%) were more likely to be found by EMS responding to an opioid overdose event at a street/highway type of location than White adults (17%).

**Table 1:** Descriptive statistics for EMS runs data collected from 2013–2017 for overall sample and by race.

	All races	%	Black race	%	White race	%
Age*						
18–25	1135	16%	136	13%	669	17%
26–35	2358	34%	297	28%	1365	35%
36–45	1458	21%	166	16%	876	23%
46–55	1079	16%	178	17%	601	16%
56–65	663	10%	197	19%	278	7%
66+	236	3%	79	8%	100	3%
Gender*						
Female	2657	38%	392	37%	1584	41%
Male	4271	62%	661	63%	2305	59%
Unknown	1	0%				
**Race**						
Asian	6	0%				
Black	1053	15%				
Hispanic	71	1%				
Other	89	1%				
Unknown	1821	26%				
White	3889	56%				
Location Type*						
Home	4513	65%	627	60%	2631	68%
Hotel/Motel	155	2%	16	2%	92	2%
Medical/Emergency/Law Enforcement/Assisted living/Nursing facility	256	4%	48	5%	145	4%
Missing	2	0%	0	0%	1	0%
Other	117	2%	23	2%	59	2%
Parking Lot	331	5%	43	4%	187	5%
Restaurant/Bar/Business	290	4%	43	4%	152	4%
Street/Highway	1265	18%	253	24%	622	16%

*Chi-square test between individuals of Black and White race indicated significant differences at p-values of <0.001 for age categories and location type and p-value<0.05 for gender.

Over time and space, the intensity of the opioid epidemic, which was measured by the kernel density plots, increased sharply from 2013 to 2017 with a noticeable increase in spatial extent from the center of the epidemic in the North, South and East direction (see S2 Fig and S2 Table). We observed differences in geographic areas where there was a high density of opioid overdose events by race (see S3 Fig, top panel) with a wider spatial extent among White adults compared to Black adults. Opioid overdose events did not vary spatially between male and female adults but more male adults than female adults suffered from an opioid overdose during the study period (see S3 Fig, bottom panel). More than 40% of overdose patients were transported to hospitals in or near the Franklinton neighborhood, including Mount Carmel West (23%) and Grant Medical Center (21%) (see S1 Table).

**Fig 1:**
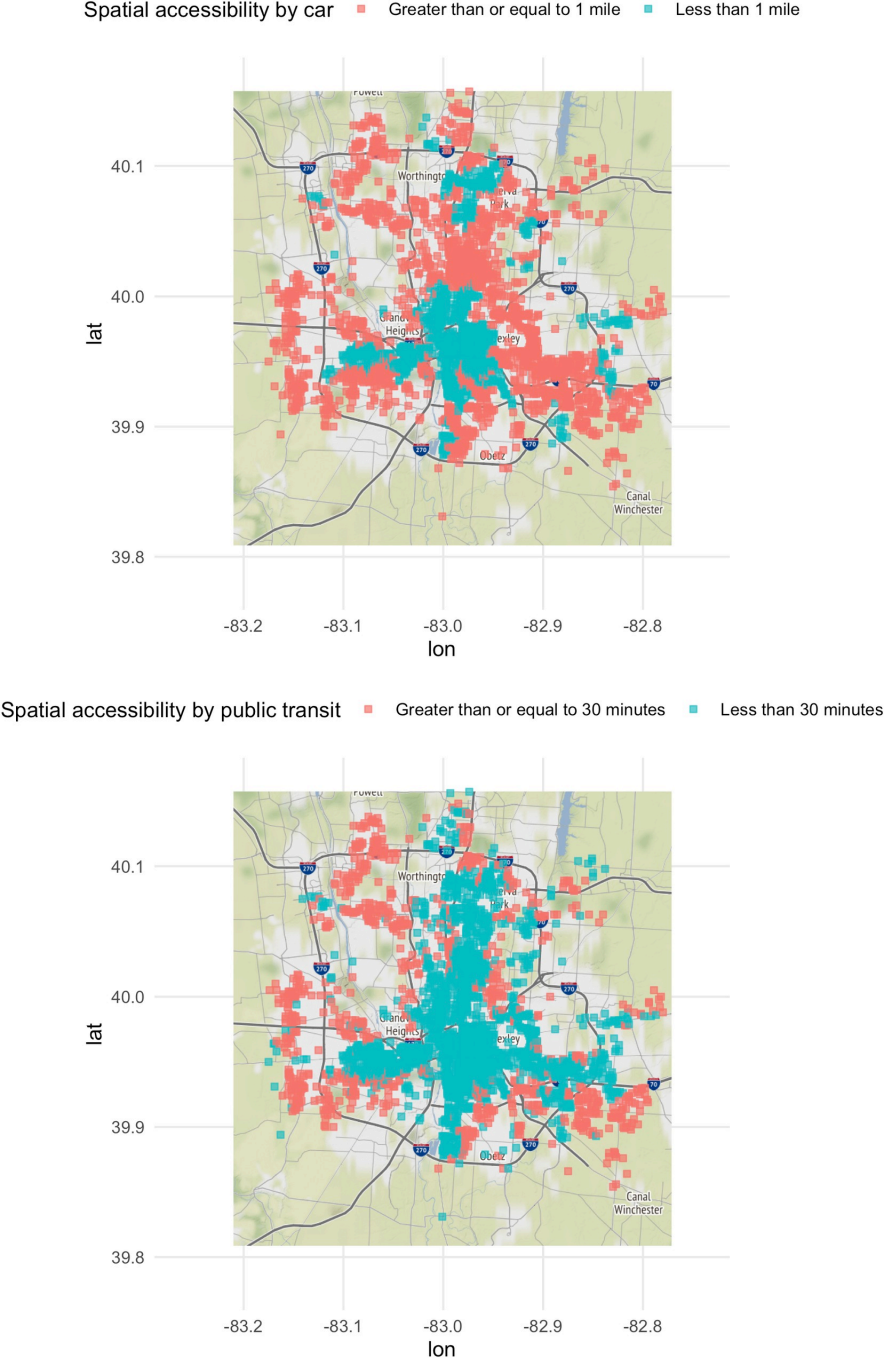
Map of study area showing Opioid Treatment Deserts (orange squares) based on travel by car using 1-mile travel distance threshold (top panel) and travel by public transit using 30-minute travel time threshold (bottom panel). Green squares are areas that are not Opioid Treatment Deserts. Black line is the Franklin County boundary. Areas without colors squares indicate that no opioid overdose events occurred in these areas based on our study’s criteria. Base map and data from OpenStreetMap and OpenStreetMap Foundation.

Opioid Treatment Deserts in Franklin County, Ohio were identified in several areas (Fig 1 for travel by car using 1-mile travel distance threshold (top panel) and for travel by public transit using 30-minutes threshold (bottom panel)). Several areas were classified as Opioid Treatment Deserts based on either mode of transportation. Areas closer to major highways and main streets and the Columbus downtown area showed greater potential spatial accessibility for OUD treatment providers. Potential spatial accessibility maps using different travel distance and time thresholds also showed urban and suburban areas that were classified as Opioid Treatment Deserts (see S4 Fig for travel by car using 2-miles travel distance threshold (top panel) for travel by public transit using a 15-minute threshold (bottom panel)). These graphs from the sensitivity analysis highlighted that there were still areas identified as Opioid Treatment Deserts when the threshold values were shifted up or down from the main results.

Only 22 providers were continuously operating from 2013 to 2017 and offered MAT for OUD (S5 Fig). These providers were located mainly in the downtown area of Columbus, OH. Median travel times were approximately 2 minutes and 17 minutes by car and public transit respectively to the closest treatment provider (Table 2). Compared to any type of MAT for OUD travel times were much higher (3 times more for travel time by car and 2.5 times more for travel time by public transit) for Methadone treatment for OUD (Table 2). Travel times were comparable for Buprenorphine and Naltrexone treatment for OUD. Travel times by were significantly different for all types of treatment providers via public transit and for Methadone treatment providers via travel by car (Table 2). Travel times were typically shorter for individuals with Black race vs White race across all modes of transportation and types of treatment providers. As a sensitivity analysis, we re-calculated travel times in Table 2 but restricting the estimation to individuals who were at a location classified as a home or residential location (this does not include nursing facilities or long-term care residential facilities). We found similar results for median travel time when limiting the analysis to home/residential type locations (Table 3) although all travel times were statistically significantly different.

**Table 2:** Travel time (in minutes) to facility by type of services offered and by mode of transportation.

		Median travel time in minutes(interquartile range = 25^th^ and 75^th^ percentile values)			Two-sided t-test for Black and White travel times	
Mode of transportation	Treatment services provided	All races	Black race	White race	t-statistic	p-value
Car	OUD*	2(1,4)	2(1,4)	2(1,4)	1.5900	0.1130
	Buprenorphine	3(2,5)	3(1,5)	3(2,5)	-0.3170	0.7510
	Methadone	6(4,8)	5(4,7)	6(5,8)	-3.6300	0.0003
	Naltrexone	3(2,5)	3(1,5)	3(2,5)	-0.3000	0.7640
Public transit	OUD*	17(9,26)	16(8,25)	17(9,26)	-3.5400	0.0004
	Buprenorphine	23(13,35)	21(11,31)	24(14,37)	-6.4300	<0.0001
	Methadone	42(26,54)	34(25,48)	44(27,55)	-8.6500	<0.0001
	Naltrexone	23(13,35)	21(11,31)	24(14,37)	-6.4000	<0.0001

*Any type of MAT

**Table 3:** Median travel time and interquartile range (25^th^ and 75^th^ quantile values) based on restricting scene location type of EMS run to Residential type only vs. any location type.

Mode of transportation	Treatment services provided	Scene location type was Residential type only	Scene location was any type of location	t-statistic	p-value
Car	OUD*	2(1,4)	2(1,4)	-2.42	0.0156
	Buprenorphine	3(2,5)	3(2,5)	-2.89	0.0038
	Methadone	6(5,8)	6(4,8)	-2.8	0.0052
	Naltrexone	3(2,5)	3(2,5)	-2.89	0.0039
Public transit	OUD*	18(10,28)	17(9,26)	-5.23	<0.0001
	Buprenorphine	25(15,37)	23(13,35)	-4.7	<0.0001
	Methadone	44(26,56)	42(26,54)	-3.5	0.0005
	Naltrexone	25(15,37)	23(13,35)	-4.69	<0.0001

*Any type of MAT

## 4. Discussion

We confirm the existence of Opioid Treatment Deserts—a geographic area with low potential spatial accessibility to treatment and recovery service providers offering evidence-based treatment (MAT) for OUD. We also confirm that potential spatial accessibility for OUD treatment differs by race travel times by car and public transit were slightly lower among Black adults compared to White adults who may seek OUD treatment. In practical terms, whether estimating spatial accessibility based on where an overdose occurs rather than place of residence holds true, we suspect that the concept of treatment accessibility constraints (e.g., in space and time) itself is valid. We believe it is valid because spatial accessibility is a known barrier to SUD treatment [12, 48, 53, 54], which includes OUD, and we were able to identify Opioid Treatment Deserts according to several different criterion (e.g., sensitivity analysis for threshold value of opioid overdose rate and travel time, Fig 1, S4 Fig). Another way to validate our findings would be using healthcare claims data that included information on place of residence of an individual seeking OUD treatment and the location of potential OUD treatment providers. Unfortunately, such an ideal dataset was not available to our team, and would also exclude those overdose events where patients are not transported, as prior to 2020 health insurances did not pay for EMS care that did not result in transport to a hospital or emergency department.

Using an EMS dataset to characterize where overdoses are occurring is novel and provides advantages to directing the opioid response locally [32, 55] yet there are challenges too given the number of assumptions we made in this study. While previous studies have identified county-level characteristics, such as lack of publicly available OUD medication providers [30], the identification of Opioid Treatment Deserts at the sub-county level may allow for targeted siting of OUD medication providers in order to reduce disparities in spatial accessibility, and, in turn, lower OUD morbidity and mortality rates. Using EMS data is an advantage in that it captured overdose events that may not require transfer to an ED. Other studies of EMS overdose data have reported 46–54% transport rates [56] although transport rates were higher in our data. EMS data also provide a verified address (the scene address), which even if it is not the patients’ actual home it may still be a location easily accessible to them, and allows us to include patients who are unhoused or unlikely to provide a true address to ED or hospital billing and registration department.

One assumption of this study was that where an opioid overdose occurred was at or near the patient’s place of residence. Opioid addiction requires use 1–4 times a day depending on the opioid, and so persons with OUD typically have to integrate using and places to use into their daily lives at work (61% are working full or part time data) and home [57]. While there is no firm evidence on the relationship of place of overdose to home residence available at this time, the nationwide quarantines, curfews, and lockdowns during the COVID-19 pandemic have not reduced opioid overdose events or deaths [58], suggesting that most people with OUD likely have a network of suppliers and use sites close to home. As the data for this study is pre-COVID pandemic, future analyses may show different trends.

Ohio saw a 13% increase in opioid related deaths May 2019-May 2020 compared to the prior year [59]. This is presumed to be from pandemic related factors, including: increased social isolation, people who are using being more likely to use alone due to social distancing guidelines, emotional and financial challenges, disrupted supply chains resulting in people using substances they do not normally use, reduced access to harm reduction measures and support systems [60]. For instance, at the height of the Ohio epidemic peak in November and December 2020, one major area hospital’s associated substance abuse treatment center was moved out to create more beds for COVID-19 infected patients. Repeating this analysis with EMS data during and (hopefully) after the pandemic could reveal increasing disparities in access to treatment.

Another assumption in our study is that all persons who experienced an overdose would want linkage to treatment or recovery services. While not all people experiencing an opioid overdose event are persons with a diagnosed substance use disorder per the Diagnostic and Statistical Manual, Version 5 criteria, an overdose event can be considered an opportunity to link those with SUD with treatment. Persons without SUD can also still benefit from secondary prevention wherein they are provided with education and other tools to help prevent a subsequent overdose (such as take home naloxone kids). All persons who experience an OUD should be screened for SUD and treatment needs.

Our findings can be placed in context of the current literature in a number of ways, Previous studies have shown that patients in recovery after a drug overdose generally travelled short distances to treatment centers and that shorter distances were associated with greater likelihood of entering and completing treatment, which does not mean treatment was successful but that the prescribed treatment regimen was completed by the individual [53, 54]. A number of studies have found that travel distance of more than 1 mile or approximately 2 minutes from place of residence to treatment provider facility by car in urban cities was associated with a lower probability of completing treatment for drug or substance abuse [12, 48, 53, 54]. More specifically of OUD, methadone treatment can require daily visits while Buprenorphine treatment commonly involves at least weekly visits. Such a travel burden could be especially prohibitive for those who depend on public transit [19], which in our study was on 17 minutes (median value) for any type of OUD treatment. Additionally, we found that individuals seeking a specific type of OUD treatment, such as Methadone treatment, may face even greater challenges due to almost double the amount of travel time to closest provider compared to other types of MAT.

Our finding of geographic areas in Columbus, OH that were Opioid Treatment Deserts irrespective of mode of transportation is alarming. We noticed these areas in the Northwest and Southeast of the city (orange squares in the top left and bottom right in Fig 1 for both modes of transportation). Some possible remedies to addressing lack of potential spatial accessibility in these areas include opening new facilities that offer MAT, mobile MAT clinics, and increasing public transit availability in these areas. While these options are likely to be costly, the burden of the opioid epidemic is nearly everywhere in Columbus, OH. Despite some areas with a higher burden of the epidemic than others, equitable access to MAT should be the main goal of public health and other agencies that are determining where to allocate limited resources.

The sensitivity analysis provided the following insights. First, while results were sensitive to the threshold value for each mode of transportation Opioid Treatment Deserts were still identified irrespective of the threshold value. Second, decision-makers can adjust the threshold value based on the personal circumstances of individuals or specific populations seeking OUD treatment.

Our finding of differences in potential spatial accessibility between racial groups may be interpreted as follows. First, even though shorter travel times were observed for the two racial group spatial accessibility is only one of several barriers to engagement in treatment [16, 61]. Racial differences of 1 minute of less are not clinically relevant for travel by car but differences of 5–10 minutes for travel by public transit (e.g., to methadone or buprenorphine treatment providers, Table 2) may have clinical relevance in terms of missing or rescheduling an appointment. Second, Methadone treatment was less spatially accessible for all (higher travel times on average compared to other types of MAT) yet for Black adults travel times by both modes of transportation were lower than for individuals with White race. In practical terms, this finding may reflect the socioeconomic disparities noted in other metropolitan areas surrounding the provision of buprenorphine and methadone. Prior evaluations suggest that methadone is preferentially provided in ethnic minority and low income areas [62, 63]. The cost of either methadone or buprenorphine treatment can be prohibitive depending on insurance status, but when it first became available in 2002 buprenorphine was 10 times the cost of methadone [64]. This led to disparities in coverage by different insurances and buprenorphine clinics being less likely to arise in poor areas. There is also high levels of stigma against methadone clinics, resulting in campaigns to block these clinics in wealthier areas [65]. Buprenorphine, which can be prescribed in a regular clinic and dispensed at any retail pharmacy, is less stigmatizing than waiting in long lines daily at a methadone clinic. Access to care for opioid treatment is shaped by decades of stigmatization and this is likely reflected in our analysis.

This is why local evaluations of where treatment providers are located and geospatial access is so important. Our analysis shows initial evidence of Opioid Treatment Deserts in our study area, specifically in regards to mode of transportation and type of MAT. These Opioid Treatment Deserts may be larger if one included the additional issue of capacity at treatment providers. Further validation and refinement of the concept of an Opioid Treatment Desert could include elements of capacity for treatment and personalized treatment planning based on the transportation options available for patients seeking treatment and recovery services. For example, only a few centers will treat pregnant patients or adolescents, creating barriers to care for these subpopulations.

Another limitation is using data from a single EMS agency over a long period of time. While using EMS data is a strength of this study, changes in practice or internal guidelines for naloxone administration, administration of naloxone to any suspected overdose based on symptomology [55] (e.g., receiving naloxone for a heart attack), how EMS responders coded opioid-related events in their database management systems, and variability between battalions (area-specific EMS teams within the Columbus Fire Department) could result in data discrepancies over the study period. These factors may introduce information bias in our own data collection efforts such that not all opioid-related overdose events were captured for all years of data collection. Another limitation of this study was the increase in availability of naloxone over the time period of data collection, which may have been associated with trends in opioid overdose events in Columbus, OH. While higher availability of naloxone has been associated with lower number of opioid overdose deaths [66] there are a number of other factors associated with dynamics of overdose events (fatal and non-fatal) that make it difficult to assess the causal relationship between naloxone availability and opioid overdose rates. These other factors include local drug market dynamics, local laws and policies, stigma and socioeconomic conditions [67]. Also, this study was conducted in a large metropolitan county and the results are not generalizable to rural counties where travel times to treatments providers is likely to be even longer. Another limitation is that we do not account for capacity of treatment providers in relation to individuals seeking OUD treatment. We did not have data on provider capacity. Therefore, we were unable to run the more traditional spatial accessibility analysis, such as the two-step floating catchment area (2SFCA) method. 2SFCA could be more suitable for areas (like downtown areas) where the competitions for accessing treatment providers maybe high because the 2SFCA method explicitly considers the supply-demand ratio (e.g, bed-to-population ratio) when computing spatial accessibility. Since we do not have information on provider capacity we believe that our approach is reasonable as an initial exploration of the concept of opioid treatment deserts. Lastly, due to the lack of freely and publicly available traffic data at road segment level, this study did not consider traffic patterns and road congestion when measuring spatial accessibility to treatment providers. Readers who want to account for the traffic effects in measuring accessibility can consult with the Google Maps API which considers the road congestion as well as the difference between peak hours versus off-peak hours.

A promising future direction in data sharing between public health and public safety is the use of real-time EMS data and up-to-date treatment provider data for allocation of limited resources for treatment and prevention of OUD and improving linkage to treatment post-overdose. This would further enhance the ability of local public health departments, regional behavioral health agencies and social service agencies to identify how to allocate or reallocate funding for harm reduction, prevention and treatment in a dynamic and responsive manner. Cross-sector data sharing capabilities, such as Health Data Exchanges, Open Data Platforms, Data Across Sectors of Health, and Smart City Operating Systems (e.g., Smart Columbus) could potentially contribute to such a future direction. Quick Response Teams could use the data up-to-date data on treatment providers to link individuals to care without worrying about the patient not being able to enter treatment because the data on OUD treatment providers was out of date, which anecdotally happens quiet often in low-resources settings even in a populous county such as Franklin County, OH. Social workers are addressing the opioid epidemic in a number of different ways, including being a part behavioral health teams [68] and Quick Response Teams [69], where one of their roles is linking individuals after an overdose with treatment and other services that will support staying in treatment. The effectiveness of social worker-based interventions could potentially be improved by optimally allocating resources in areas identified as Opioid Treatment Deserts. The concept of Opioid Treatment Deserts could be further extended to other dimensions of access-related barriers to healthcare (e.g., availability, affordability, accommodation, accessibility, and acceptability) in order to identify treatment deserts for many other clinical and public health outcomes where repeated or frequent access to services is an integral part of treatment/prevention efforts or minimizing risk of re-admission after a hospital admission or encounter. Examples of such outcomes include, other types of addiction-related diseases, follow-up care after a medical procedure, transition care, prenatal care, pediatric care, and pain management.

In conclusion, Opioid Treatment Deserts are geographic areas with low potential spatial accessibility to treatment and recovery service providers offering evidence-based treatment (MAT) for OUD. Although further studies are needed to verify and validate the concept of an Opioid Treatment Desert, our results suggest not only that there are space-time barriers to accessing treatment for OUD but, furthermore, provides direct information on where in space such disparities exist in terms of insufficient supply of treatment providers. Addressing accessibility overall and inequalities in accessibility over space may also be critically important to successfully implementing the Cascades of Care Model for OUD, which highlights the need for a holistic approach to prevention and treatment for OUD patients seeking treatment and staying in remission [6]. Increasing retention rates in OUD treatment is another likely factor that may be affected by allocating resources in Opioid Recovery Deserts because multiple barriers to access have been identified as affected retention rates including travel time [12, 70]. These implications of the Opioid Treatment Desert concept have the potential to reduce the burden of the opioid epidemic in the US through data-driven and contextually relevant approaches as exemplified in this study.

## Supporting information

S1 Fig Map showing Emergency Medical Services (EMS) jurisdiction area (in color) served by Columbus Fire Department. (TIF)

S2 Fig Heat map of spatial and temporal pattern of opioid overdose events in Franklin County. Note that Columbus Fire Department from 2008 to 2017 but main analysis included data from 2013–2017, inclusive. Base map and data from OpenStreetMap and OpenStreetMap Foundation.
(TIF)

S3 Fig Heatmaps of overdose events by gender and race in Columbus Fire Department jurisdiction based on data from 2013–2017. Base map and data from OpenStreetMap and OpenStreetMap Foundation.
(TIF)

S4 Fig **Map of study area showing Opioid Treatment Deserts (orange squares) based on travel by car using 2-miles travel distance threshold (top panel) and travel by public transit using 15-minute travel time threshold (bottom panel).** Green squares are areas that are not Opioid Treatment Deserts. Areas without colors squares indicate that no opioid overdose events occurred in these areas based on our study’s criteria. Base map and data from OpenStreetMap and OpenStreetMap Foundation.
(TIF)

S5 Fig Map of study area showing OUD treatment providers (green triangles), City of Columbus boundary (shaded gray areas), and major roads/highways. (TIF)

S1 Table Hospital destination for all opioid overdose patients in Columbus Fire Department service area from 2013 to 2017. (DOCX)

S2 Table Annual number of opioid overdose patients in Columbus Fire Department service area. Main analysis was restricted to data from 2013 to 2017, inclusive.
(DOCX)

S1 Data (R)
