# Supplementary figures and images for: Opioid Treatment Deserts: Concept development and application in a US Midwestern urban county
Source: PLoS One. 2021 May 12;16(5):e0250324. doi: 10.1371/journal.pone.0250324 (PMC8115812; doi:10.1371/journal.pone.0250324)

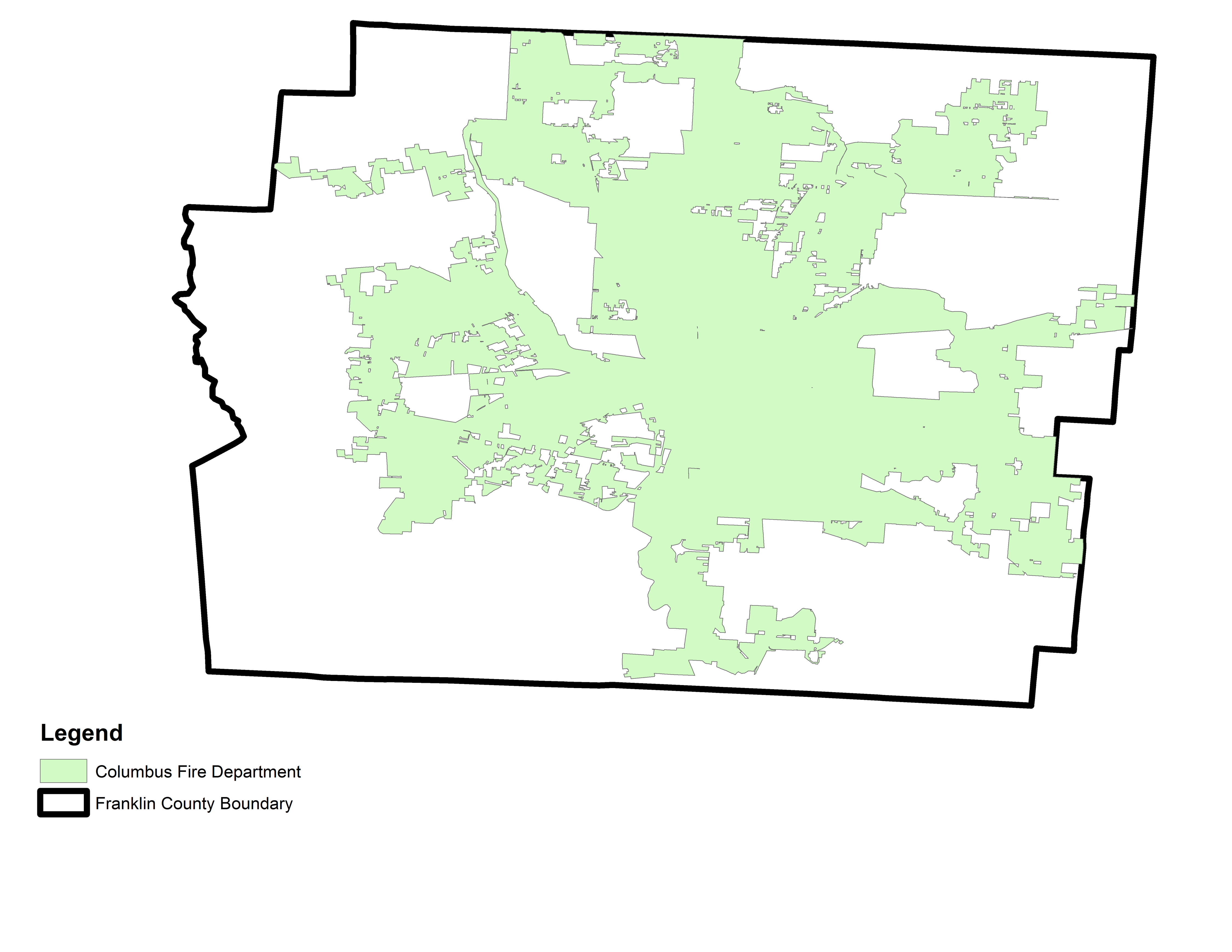

Supplement: S1 Fig — (TIF) [file pone.0250324.s001.tif]

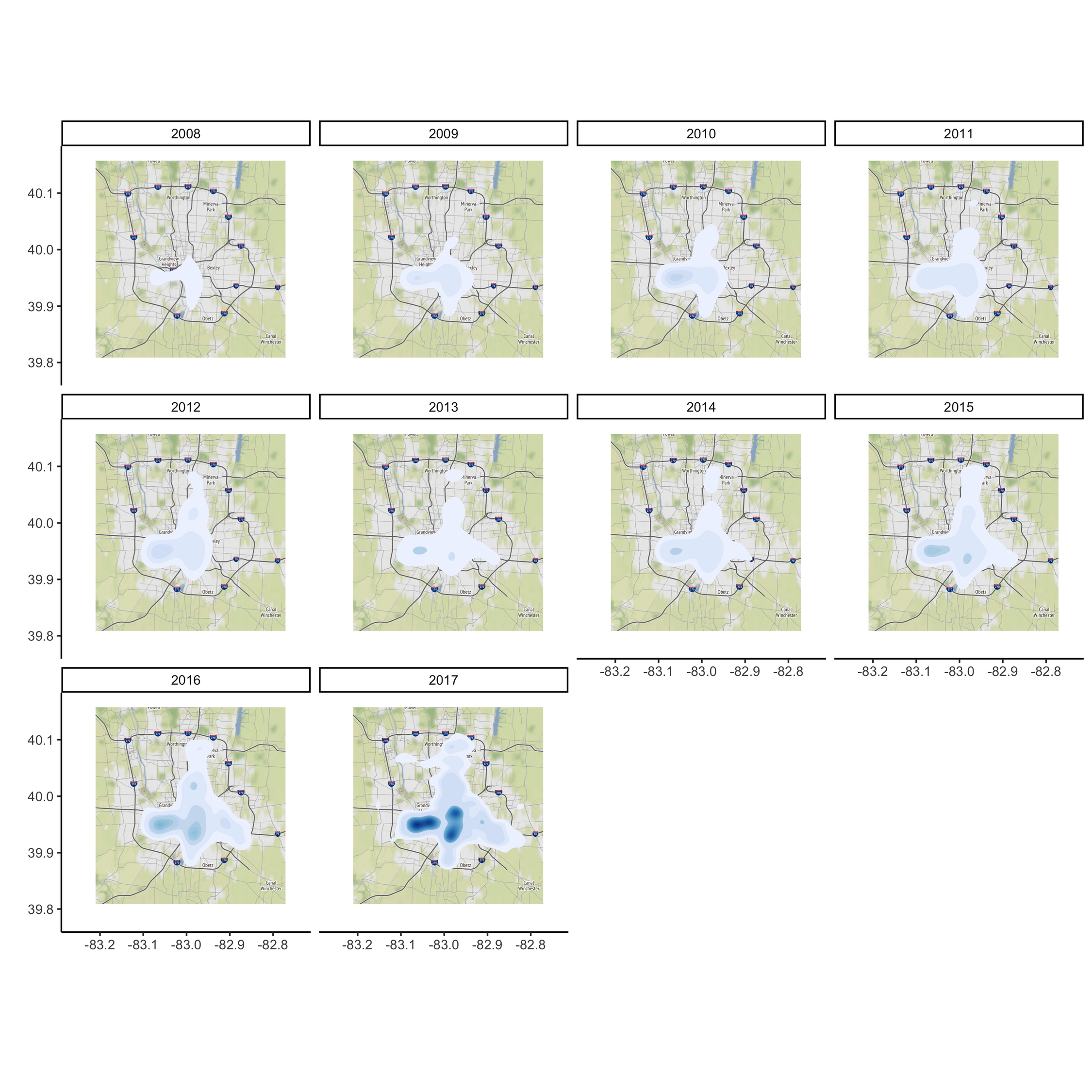

Supplement: S2 Fig — Note that Columbus Fire Department from 2008 to 2017 but main analysis included data from 2013–2017, inclusive. Base map and data from OpenStreetMap and OpenStreetMap Foundation. (TIF) [file pone.0250324.s002.tif]

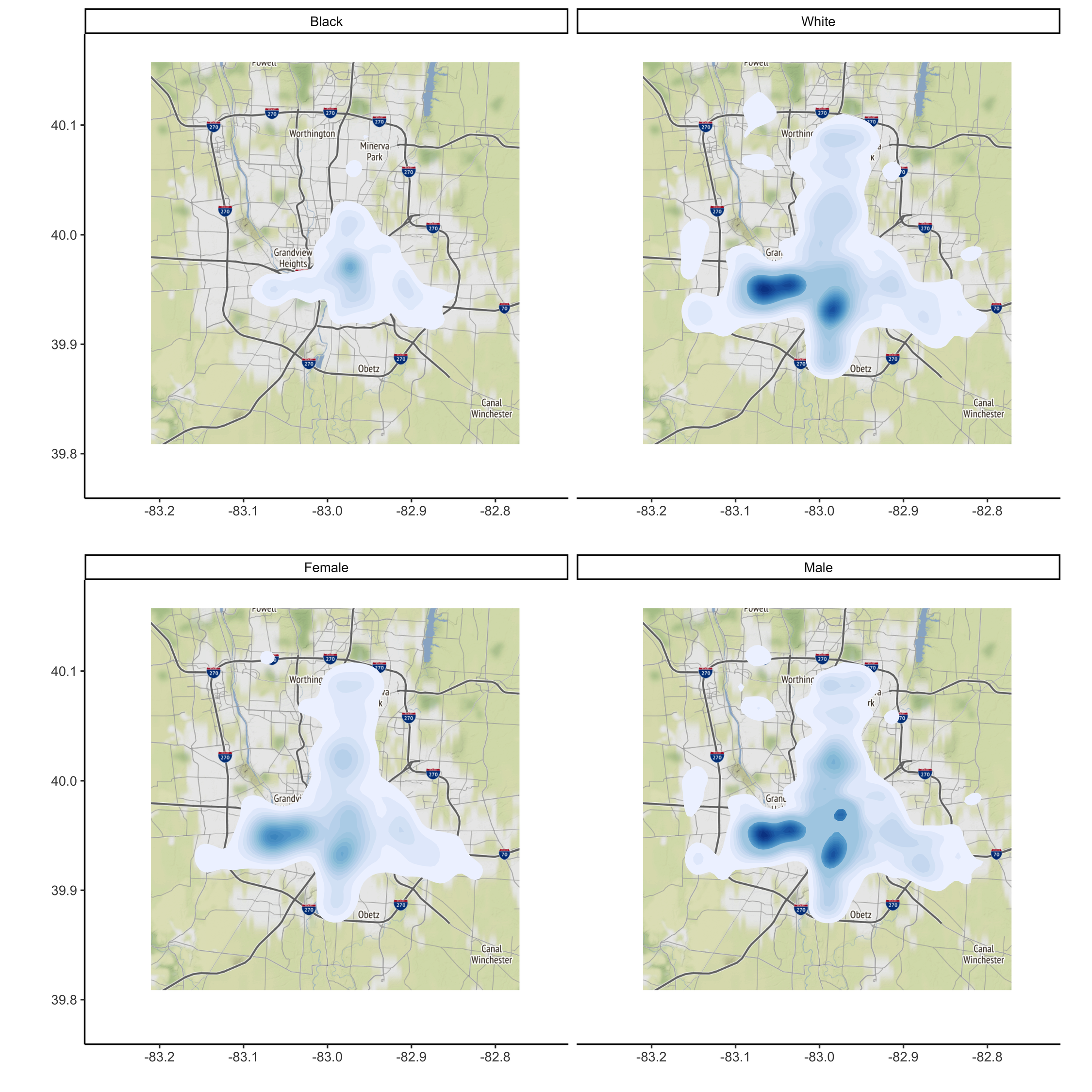

Supplement: S3 Fig — Base map and data from OpenStreetMap and OpenStreetMap Foundation. (TIF) [file pone.0250324.s003.tif]

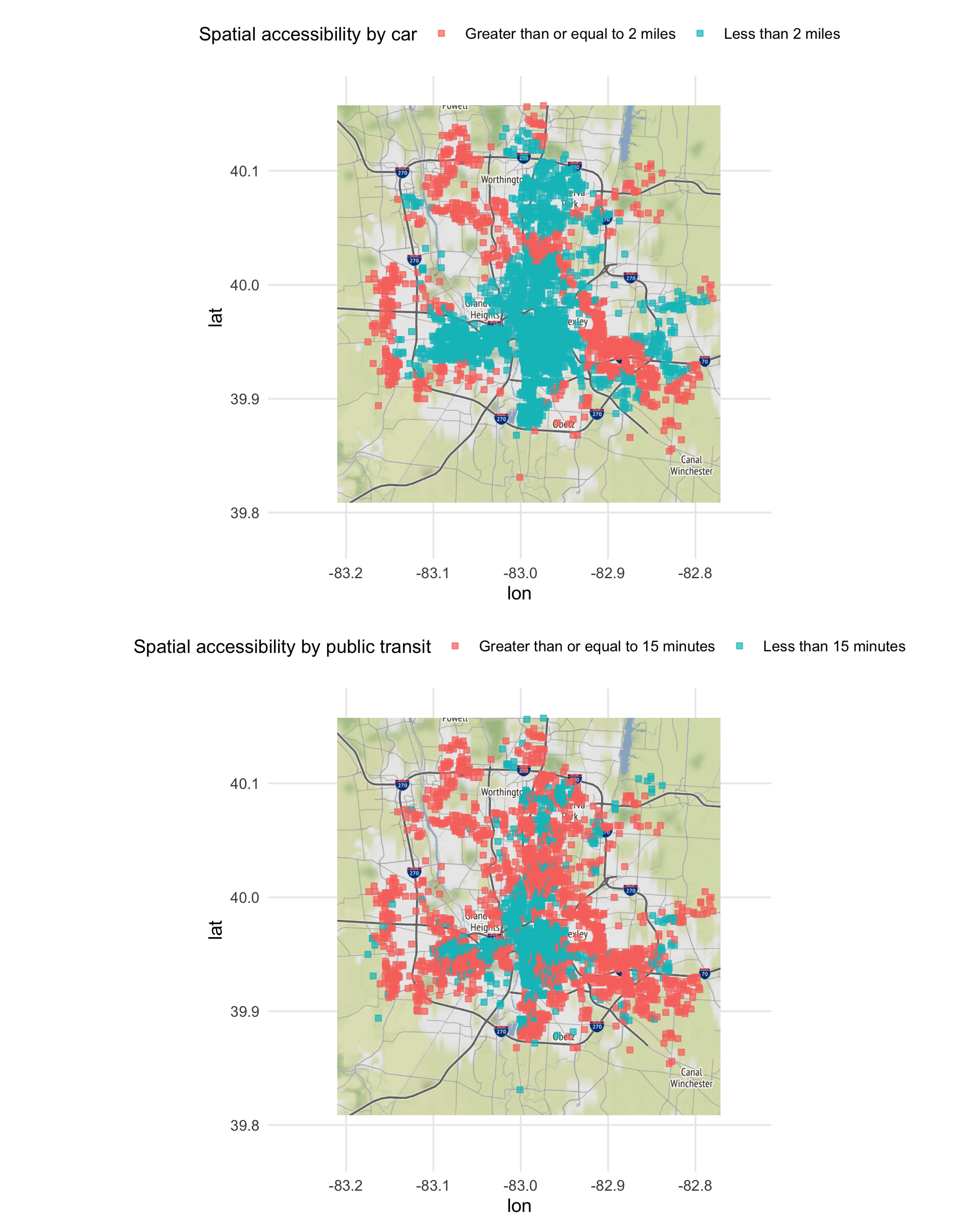

Supplement: S4 Fig — Map of study area showing Opioid Treatment Deserts (orange squares) based on travel by car using 2-miles travel distance threshold (top panel) and travel by public transit using 15-minute travel time threshold (bottom panel). Green squares are areas that are not Opioid Treatment Deserts. Areas without colors squares indicate that no opioid overdose events occurred in these areas based on our study’s criteria. Base map and data from OpenStreetMap and OpenStreetMap Foundation. (TIF) [file pone.0250324.s004.tif]

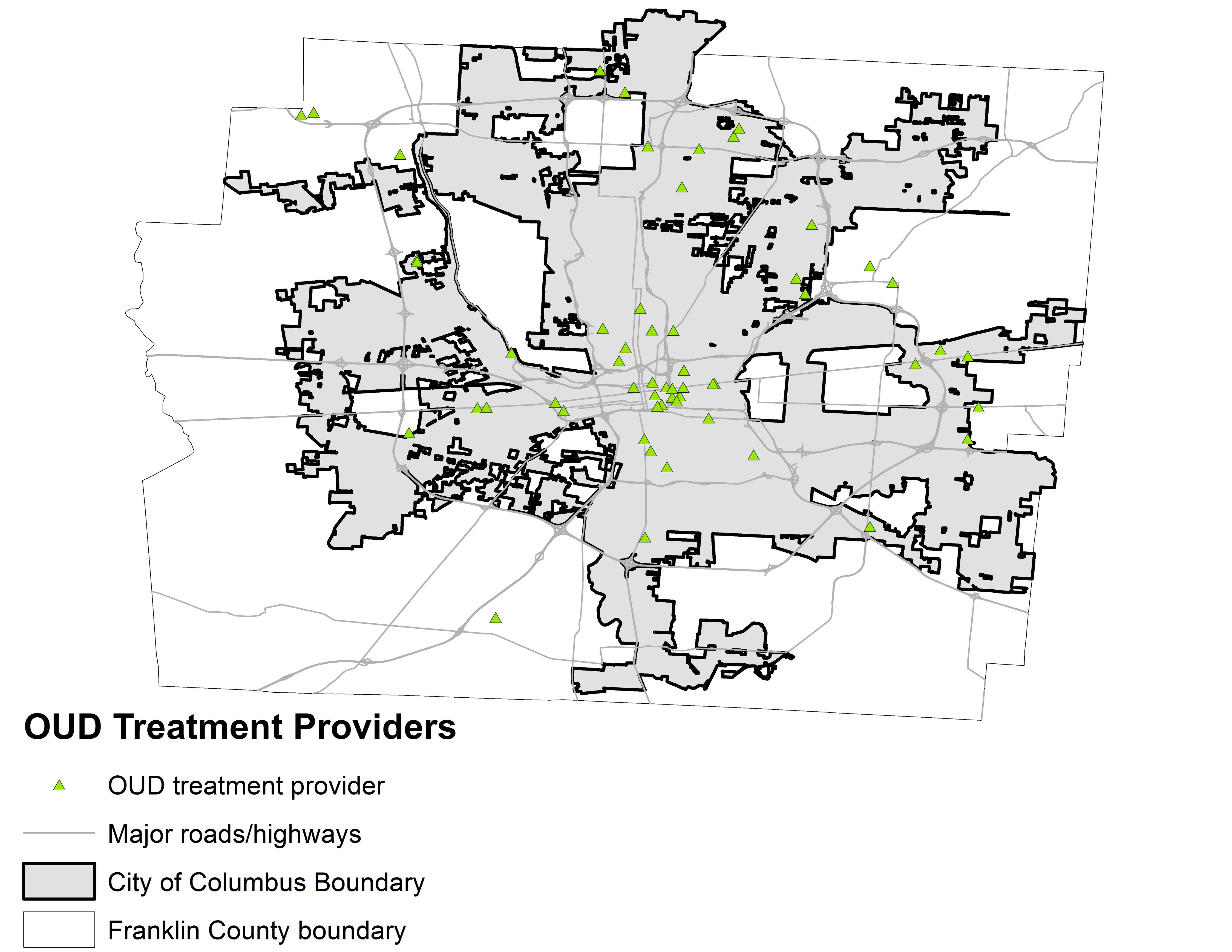

Supplement: S5 Fig — (TIF) [file pone.0250324.s005.tif]
