# Supplementary material for: Opioid Treatment Deserts: Concept development and application in a US Midwestern urban county
Source: PLoS One. 2021 May 12;16(5):e0250324. doi: 10.1371/journal.pone.0250324 (PMC8115812; doi:10.1371/journal.pone.0250324)
Supplement: S1 Table — (DOCX) [file pone.0250324.s006.docx]

**S1 Table.** Hospital destination for all opioid overdose patients in Columbus Fire Department service area from 2013 to 2017.

| **Hospital** | **N** | **%** |
| --- | --- | --- |
| Doctor's Hospital | 351 | 5% |
| Dublin Methodist | 38 | 1% |
| Grant Medical Center | 1430 | 21% |
| Mount Carmel East | 493 | 7% |
| Mount Carmel St. Ann’s | 481 | 7% |
| Mount Carmel West | 1599 | 23% |
| Nationwide Children’s Hospital | 2 | 0% |
| No transport | 8 | 0% |
| OSU East | 839 | 12% |
| OSU Wexner Medical Center | 288 | 4% |
| Other | 34 | 0% |
| Riverside Methodist | 721 | 10% |
| Unknown | 645 | 9% |
