# Supplementary material for: Opioid Treatment Deserts: Concept development and application in a US Midwestern urban county
Source: PLoS One. 2021 May 12;16(5):e0250324. doi: 10.1371/journal.pone.0250324 (PMC8115812; doi:10.1371/journal.pone.0250324)
Supplement: S2 Table — Main analysis was restricted to data from 2013 to 2017, inclusive. (DOCX) [file pone.0250324.s007.docx]

**S2 Table.** Annual number of opioid overdose patients in Columbus Fire Department service area. Main analysis was restricted to data from 2013 to 2017, inclusive.

| **Year** | **Number of EMS runs for opioid overdose events** | **%** |
| --- | --- | --- |
| 2008 | 395 | 4% |
| 2009 | 593 | 6% |
| 2010 | 721 | 7% |
| 2011 | 823 | 8% |
| 2012 | 880 | 9% |
| 2013 | 859 | 8% |
| 2014 | 916 | 9% |
| 2015 | 1085 | 11% |
| 2016 | 1469 | 14% |
| 2017 | 2600 | 25% |
